# Enhanced accumulation of reduced glutathione by Scopoletin improves survivability of dopaminergic neurons in Parkinson’s model

**DOI:** 10.1038/s41419-020-02942-8

**Published:** 2020-09-10

**Authors:** Priyadarshika Pradhan, Olivia Majhi, Abhijit Biswas, Vinod Kumar Joshi, Devanjan Sinha

**Affiliations:** 1 Department of Zoology, Institute of Science, Banaras Hindu University, Varanasi, 221005 India; 2 Department of Dravyaguna, Institute of Medical Sciences, Banaras Hindu University, Varanasi, 221005 India

**Keywords:** Parkinson's disease, Parkinson's disease

## Abstract

Parkinson’s disease (PD) is a neuromotor disorder, primarily manifested by motor anomalies due to progressive loss of dopaminergic neurons. Although alterations in genetic factors have been linked with its etiology, exponential accumulation of environmental entities such as reactive oxygen species (ROS) initiate a cyclic chain reaction resulting in accumulation of cellular inclusions, dysfunctional mitochondria, and overwhelming of antioxidant machinery, thus accelerating disease pathogenesis. Involvement of oxidative stress in PD is further substantiated through ROS induced Parkinsonian models and elevated oxidative markers in clinical PD samples; thereby, making modulation of neuronal oxidative load as one of the major approaches in management of PD. Here we have found a potent antioxidant moiety Scopoletin (Sp), a common derivative in most of the nootropic herbs, with robust neuroprotective ability. Sp increased cellular resistance to ROS through efficient recycling of GSH to prevent oxidative damage. The Sp treated cells showed higher loads of reduced glutathione making them resistant to perturbation of antioxidant machinery or neurotoxin MPP^+^. Sp could restore the redox balance, mitochondrial function, and prevented oxidative damage, leading to recovery of dopaminergic neural networks and motion abilities in *Drosophila* genetic model of PD. Our data also suggest that Sp, in combination increases the therapeutic potency of L-DOPA by mitigating its chronic toxicity. Together, we highlight the possible ability of Sp in preventing oxidative stress mediated loss of dopaminergic neurons and at the same time enhance the efficacy of dopamine recharging regimens.

## Introduction

Parkinson’s disease (PD) is a progressive neurological disorder manifested clinically in four cardinal motor symptoms; muscular rigidity, bradykinesia, resting tremor, and postural imbalance occurring at later stages^1^. These chronic attributes are generally associated with selective degeneration of dopaminergic neurons, corollary de-pigmentation of substantia nigra pars compacta (SNpc) and concomitant accumulation of α-synuclein in insoluble inclusions called Lewy bodies^2,3^. Most of PD cases are sporadic and only a minor subset of overall PD cases is associated to be familial^4,5^. Although α-synuclein is frequently implicated in PD pathogenesis, other proteins such as PTEN induced putative kinase 1 (PINK1), Parkin, DJ-1, leucine-rich repeat kinase 2 (LRRK2) have been strongly linked to familial forms of PD^6,7^.

Given the progressive nature of the disorder, disease pathogenesis is mainly triggered by continual processes such as geometric accumulation of oxidative radicals brought about by cellular inclusions and mitochondrial dysfunction^8–10^. For example, PINK1 and Parkin loss of function impair mitophagy and cause accumulation of damaged mitochondria, leading to enhanced electron leakage and formation of superoxides^11–13^. Similarly, α-synuclein oligomers intervene with organellar functions and interact with metal ions to cause oxidative stress^14^. Mitochondrial abnormalities have also been associated with point mutations in Hsp70 chaperone that makes it aggregation prone, thereby, increasing iron and free-radical load^15–17^. Therefore, accelerated oxidation of biomolecules acts both as a product and causal for disease manifestation which is further exacerbated by failure of antioxidant system and loss of redox sensing capabilities of chaperone DJ-1^18,19^.

The association of ROS with neuronal degeneration in PD is further substantiated by modeling the Parkinsonian phenotypes in animals through 1-methyl-4-phenyl-1,2,3,6-tetrahydropyridine (MPTP), rotenone, 1,1′-dimethyl-4,4′-bipyridinium dichloride (paraquat), or 6-hydroxydopamine (6-OHDA), those are known of elevate the oxygen free-radicals in the cell^18^. Substantial number of evidences proposes ROS to be a major contributor of dopaminergic neuronal loss in the PD brain. High ROS in SNpc neurons usually results from dopamine metabolism, low glutathione, and high levels of iron and calcium^20^. Dopamine is an unstable molecule that undergoes auto-oxidation or oxidative deamination through enzyme monoamine oxidases to generate peroxides^21^. In addition, presence of high concentrations of polyunsaturated fatty acids in the brain enhances their peroxidation and generation of toxic products which further causes degeneration of the cells harboring them or in vicinity^22^.

The contribution of oxidation in PD pathogenesis has also been supported through clinical studies reporting alterations in serum glutathione peroxidase and superoxide dismutase activities, together with high malondialdehyde (MDA) levels in PD patients^23,24^. Recent metanalysis have revealed significant increase in oxidative stress markers such as MDA and a substantial depletion of antioxidants such as catalase, GSH in the blood, and postmortem brain of PD patients compared to healthy controls^25^. Therefore, regardless the clinical heterogeneity or underpinning genetics, the different forms of PD share a common etiology in mitochondrial dysfunction and oxidative stress.

Therefore, targeting cellular oxidative stress through antioxidants has been suggested as a potential therapeutic option against neurological disorders, particularly using natural plant extracts. Several studies have demonstrated effectiveness of different phytoderivatives in retracting disease pathogenesis, under various in vitro and in vivo models of PD^26^. In *Drosophila* MPTP probenecid model, mulberry fruit extracts were found to reduce nucleation of alpha-synuclein^27^. Similar results were obtained through Oleuropein derivatives from olive fruit extracts, which prevented α-synuclein fibrillation and oligomer toxicity^28^. Extracts from *Decalepis hamiltonii* delayed early onset of PD symptoms, improved circadian rhythm and motor activity in PD model flies possessing A30P and A53T α-synuclein mutation which are known to cause accelerated age dependent deterioration of circadian rhythm^29^. The extract also safeguarded the flies against paraquat induced oxidative stress and enhanced antioxidant defense^29^. Protection against paraquat treatment, locomotor impairment, shortened lifespan, and increased lipid peroxidation was observed in *parkin*RNAi flies treated with *Persea americana* peel extracts^30^. Administration of PINK1^B9^ flies with *Mucuna puriens* extracts resulted in increased GSH amount and SOD activity, together with restoration of mitochondrial morphology in crop muscles, olfactory response, climbing behavior, extended lifespan, and delay in L-DOPA induced long term motor complication^31–33^. However, the limited translation of these extracts into effective response in clinical trials, underlined further elucidation of potential candidates.

In the present study, we have reported a potent neuroprotective agent possessing robust antioxidant properties, isolated from *Convolvulus pluricaulis*, traditionally used as brain stimulator, memory enhancer and for mental debility. The presence of Scopoletin (Sp) is not limited to *C. pluricaulis* and characterized in many mental health promoting nootropic herbs such as *Evolvulus alsinoides*^34,35^. Sp could efficiently restore redox balance in cells depleted for antioxidant properties or with dysfunctional mitochondria, mainly by enhancing the reducing potential of the cell through efficient recycling of GSH. The cytoprotective properties of Sp extended to decreased progression of degenerative phenotypes in *Drosophila* Parkinson model due to retention of redox balance in dopaminergic neurons and maintenance of their architecture. Sp could also increase the efficacy of L-DOPA, extensively used in palliative care of PD patients, by subsidence of its long term toxicity; highlighting its potential development as neuroprotective agent.

## Results

### Scopoletin enhances antioxidant potential through replenishment of reduced glutathione

**Fig. 1:**
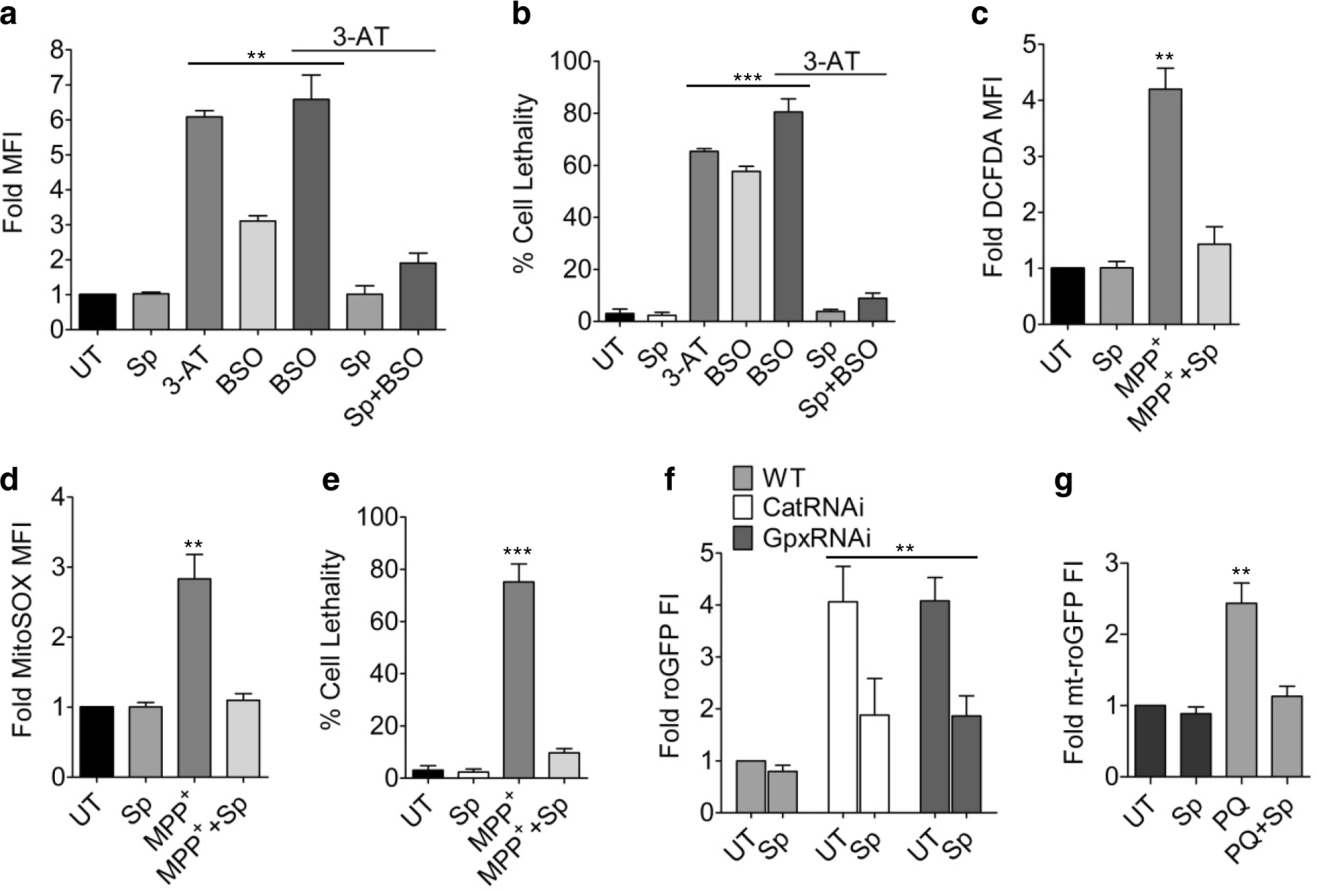
Sp promotes redox-balance and cell survivability under oxidative stress and mitochondrial dysfunction. **a** The ROS scavenging property of Sp was tested by exposing HeLa cells to 10 µM 3-AT to generate intrinsic oxidative radicals, followed by 250 µM Sp treatment for 15 min and staining with DCFDA. 50 µM BSO was used to deplete cellular glutathione. Mean DCFDA-fluorescence intensity (MFI) is represented as fold change over untreated samples. Data depicts mean ± s.e.m. *n* = 3, **P (*t*-test) <0.001. **b** The protection of the stressed cells from ROS induced cell death by 250 µM Sp treatment (for 15 min), was monitored through MTT assay and represented as mean ± s.e.m.; *n* = 8, ***P (*t*-test) <0.0001. **c**, **d** Mitochondrial dysfunction and oxidative stress was generated by exposing SHSY-5Y cells to 1 mM MPP^+^ for 24 h. The cells were then treated with 250 µM Sp for 15 min, stained by DCFDA (**c**) or MitoSOX (**d**) and MFI was represented as fold change over untreated cells. Bars show mean ± s.e.m. *n* = 3, **P (*t*-test) <0.001. **e** SHSY-5Y cells were subjected to treatment regimen as mentioned in (**c**) and the viability of cells were measured through MTT assay and depicted as mean ± s.e.m.; *n* = 8, ***P (*t*-test) <0.0001. **f** Sp reduces neuronal ROS in *Drosophila*. Relative fluorescence intensities (FI) of genetically expressing roGFP (redox-sensitive GFP) in fly brains depleted for Catalase or Glutathione peroxidase, untreated or treated with 2.5 mM Sp, compared to untreated wild-type controls. Bars represent mean ± s.e.m.; *n* = 10, **P (*t*-test) <0.001. **g** The potency of Sp in maintaining mitochondrial redox balance in paraquat-induced oxidative stress model was tested by exposing *Oregon R*^*+*^ *Drosophila* flies to 10 mM paraquat (PQ), followed by 24 h co-treatment with 2.5 mM Sp. Bars represent fold change of mt-roGFP fluorescence intensity (FI) over untreated flies, as mean ± s.e.m.; *n* = 10, **P (*t*-test) <0.001. UT – untreated with Sp.

**Fig. 2:**
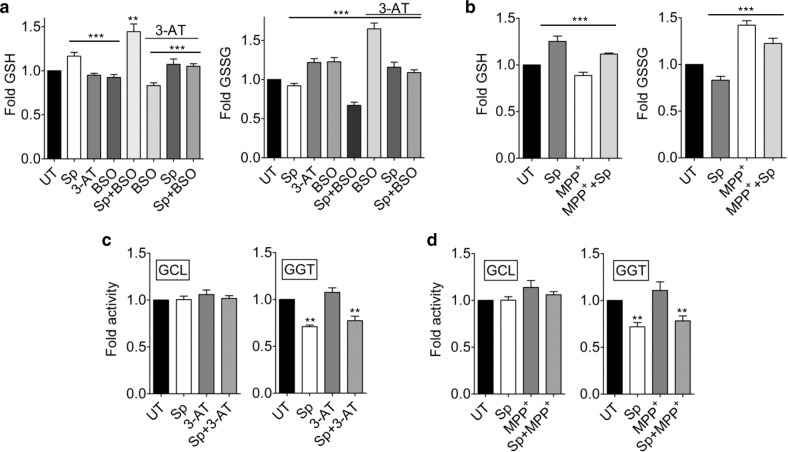
Sp recycles cellular reduced glutathione. The levels of cellular GSH or GSSG was quantified in HeLa cells treated with 10 µM 3-AT and 50 µM BSO for 15 min (**a**) or SHSY-5Y cells administered with 1 mM MPP^+^ for 24 h (**b**), along with 250 µM Sp using GSH/GSSG-Glo kit. Data are plotted as fold change over untreated controls and depict mean ± s.e.m.; *n* = 3, ***P (*t*-test) <0.0001. **c**, **d** Estimation of GCL and GGT activities in HeLa (**c**) and SHSY-5Y (**d**) cells subjected to treatment protocols as mentioned above. The activities determined as units/µg of protein has been depicted relative to the untreated controls. Bars show mean ± s.e.m.; *n* = 3, **P (*t*-test) <0.001. UT–untreated with Sp.

The ability of Sp to combat intracellular oxidative stress was tested by inhibiting Catalase by 3-amino,1,2,4 triazole (3-AT), followed by treatment with varying concentrations of the compound. The resultant elevation of oxidative radicals was found to be significantly reduced in cells further exposed to 250 µM Sp (Fig. 1a, Supplementary Fig. S1b). The Sp treated cells recovered completely from stress induced cell death observed in case of 3-AT (Fig. 1b, Supplementary Fig. S1c, d), highlighting its role as a potential antioxidant and possible moiety associated with neuroprotective behavior of many nootropic herbs^34^. Indeed, Sp was able to restore cytosolic and mitochondrial redox balance in neuronal cell line SHSY-5Y subjected to 1-methyl-4-phenylpyridinium (MPP^+^) treatment which is known to cause mitochondria dysfunction and oxidative stress through complex I inhibition^1^ (Fig. 1c, d). Hence, Sp treated neuronal cells showed enhanced survivability even after MPP^+^ exposure (Fig. 1e). The Sp, by itself was found to be nontoxic at even higher concentrations (Supplementary Fig. S1a). The ROS scavenging property of Sp was also replicated in the *Drosophila* models knocked-down for essential antioxidant enzymes catalase and glutathione peroxidase, where neuronal cells of 15 days AEL flies genetically expressing a redox biosensor, roGFP showed reduced fluorescence under Sp treatment (Fig. 1f). A parallel phenotype was observed in paraquat-treated fly model where Sp administration resulted in lower accumulation of oxygen radicals in mitochondria of brain neurons (Fig. 1g). The antioxidant property of Sp was however, found to be insensitive to depletion of glutathione by BSO, which generally causes loss of cellular antioxidant function and higher ROS (Fig. 1a). Sp treatment considerably reduced ROS and promoted higher cell survivability in BSO treated cells, in presence or absence of 3-AT, mainly by maintaining the availability of reduced glutathione (Fig. 1a, b). The cells treated with Sp showed slight enhancement in levels of GSH (~1.2 fold), together with minor alterations in GSSG levels, compared to untreated samples which exhibited higher GSSG content, thus indicating efficient recycling of GSSG to GSH (Fig. 2a). The idea was further validated by inhibiting glutathione synthesis by BSO where Sp administration caused GSH to increase by ~1.5 fold with concomitant reduction in GSSG (Fig. 2a). The potency of Sp in maintaining the reducing equivalent was further reflected in superior GSH/GSSG ratio (approx. two fold) under BSO exposure in presence of 3-AT, and lower GSSG in BSO treated cells uninhibited for catalase (Fig. 2a). The capability of Sp to replenish reduced glutathione levels was further tested in neuronal cells exposed to MPP^+^, the toxic bioactivation product of MPTP known to induce Parkinsonian phenotype in animal models^1^. The levels of GSH was slightly reduced in MPP^+^-treated SHSY-5Y cells, which was further restored to baseline levels by Sp, together with accompanying decrease in accumulated GSSG (Fig. 2b). The observed accumulation of GSH in Sp treated cells was probably due to reduced activity of γ-glutamyl transpeptidase (GGT), an enzyme responsible for glutathione catabolism^36^. Both HeLa and SHSY-5Y cells showed comparatively lower GGT activity upon Sp treatment, although the activities of glutamate–cysteine ligase were largely comparable (Fig. 2c, d). This indicates that antioxidant property of Sp encompasses replenishment of intracellular glutathione, probably through inhibition of its breakdown; thereby, making the cell more resistant to intrinsic or extraneous stressors.

### Amelioration of degenerative phenotypes in *park*^13^ dopaminergic neurons

**Fig. 3:**
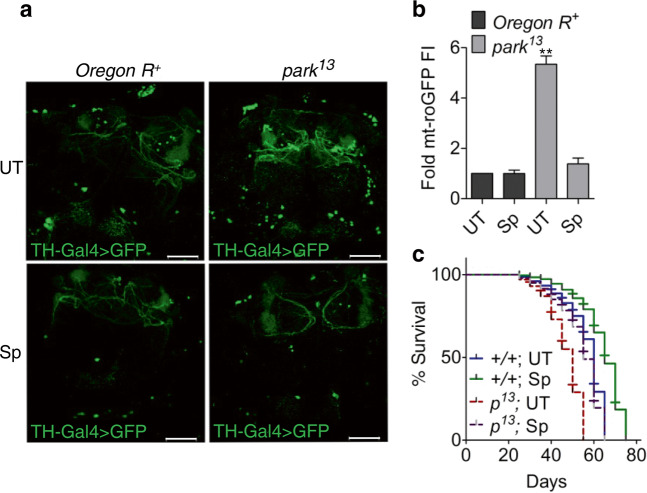
Sp promotes recovery of dopaminergic neurons in *park* flies. **a** Confocal images of *Drosophila* brains showing architecture of dopaminergic neurons labeled with GFP under wild type and mutant backgrounds, left untreated or treated with Sp. Scale bar: 50 µm. **b** The fluorescence intensity (FI) profile of *park*^13^ or wild-type *Drosophila* dopaminergic neurons expressing mt-roGFP under 2.5 mM Sp treated or untreated conditions. Data is represented as fold difference over untreated wild-type control. Bars denote mean ± s.e.m.; *n* = 3, **P (*t*-test) <0.001. **c** Kaplan Meier curve representing survivability pattern for wild type and *park*^*13*^ (*p*^13^) flies untreated or treated continuously with 2.5 mM Sp; ***P (Mantel-Cox test) <0.0001. UT – untreated with Sp.

Oxidative stress is one of the major underlying causes of sporadic and familial forms of Parkinson’s disease and mainly characterized by an imbalanced GSH/GSSG cycle, enhancing cellular susceptibility to oxidation^37^. The maintenance of glutathione cycle and survivability in neurotoxin exposed cells by Sp, led us to test its plausibility in recovering the degenerative phenotypes of well-established *Drosophila* Parkinson mutant model *park*^13^. The dopaminergic neurons of *park*^13^ and wild type control flies were marked with TH-Gal4 driven expression of UAS-GFP. Imaging of 20 days adult flies showed the neuronal architecture of Sp treated *park*^13^ flies similar to the wild type controls whereas their untreated counterparts showed shrinkage, aggregation, and complete disruption of dopaminergic neural networks (Fig. 3a). Expression of mt-roGFP in *park*^13^ tyrosine hydroxylase positive neurons showed significantly reduced mitochondrial ROS accumulation under Sp treatment as compared to untreated ones, highlighting potential mitochondrial recovery and prevention of oxidation induced cell death in dopamine producing neurons (Fig. 3b). Recovery from the degenerative phenotypes and reduced oxidative stress therefore, contributed to better survivability of the Sp-*park*^13^ flies, which was comparable to *Oregon R*^*+*^ (Fig. 3c). Sp treated wild-type flies though presented a better life span than untreated controls, did not show any significant difference in their oxidative profile and neural organization; nor were found susceptible to extraneous stressors, highlighting that Sp exposure did not compromise organismal response to electrophiles (Fig. 3c, Supplementary Fig. S1e).

### Prevention of oxidative damage and maintenance of mitochondrial function

**Fig. 4:**
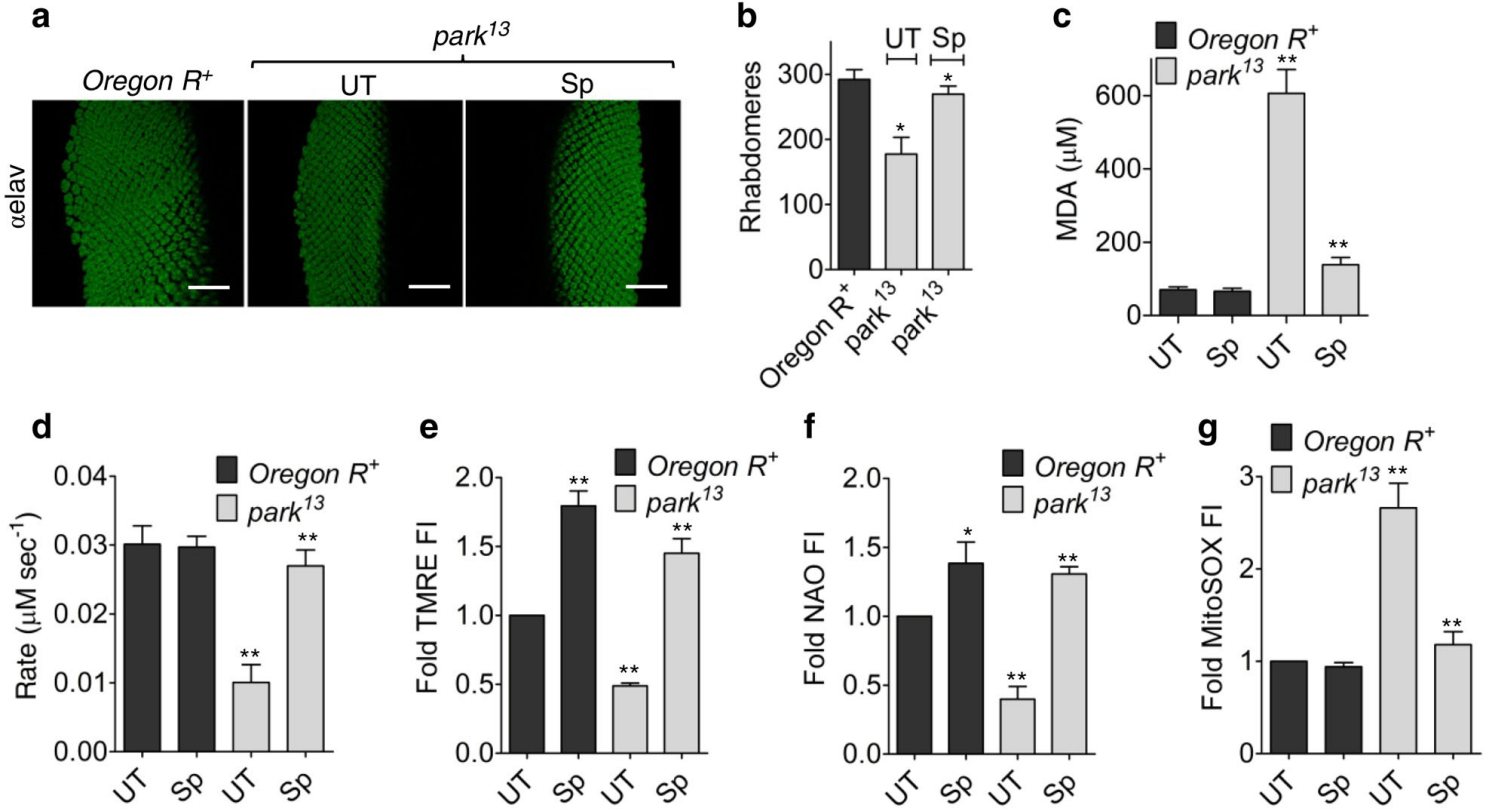
Sp prevents oxidative cellular and mitochondrial damage. **a**, **b** Eye imaginal disks derived from wild type or *park*^*13*^ third instar larvae, stained with anti-elav antibody to label the rhabdomeres and imaged with confocal microscope. Scale bar: 30 µm. The number of rhabdomeres was counted and represented as mean ± s.e.m.; *n* = 3, *P (*t*-test) <0.01 (**b**). **c** The extent of lipid peroxidation in 2.5 mM Sp treated/untreated wild type or mutant brains was measured by quantifying the levels of malondialdehyde (MDA) through thiobarbituric acid reactive substances assay. **d** The respiratory complex I activity was measured by observing the decrease in absorbance at 340 nm due to oxidation of NADH. Bars represent mean ± s.e.m.; *n* = 3, **P (*t*-test) <0.001. **e** The maintenance of mitochondrial membrane potential in *park*^*13*^ flies was measured by retention of TMRE dye in neuronal mitochondria and represented as fold change over untreated wild type. **f** The accumulation of mitochondrial mass in neurons of fly brain due to Sp treatment was determined by measuring the relative uptake N-Nonyl Acridine Orange (NAO) dye over the untreated samples. **g** The levels of mitochondrial superoxides in respective fly brains were estimated through quantification of MitoSOX fluorescence intensity (FI) and denoted as fold change over untreated wild type. All data are represented as mean ± s.e.m.; *n* = 10, **P (*t*-test) <0.001, *P (*t*-test) < 0.01.

The neuroprotective behavior displayed by Sp intrigued a parallel study in the *Drosophila* eye since it displays detectable phenotypes in response to neurophysiological disruptions such as progressive loss of ommatidia accompanied with disrupted rhabdomere arrangement^38^. Immunohistochemical analysis of eye imaginal disks from 120 h AEL third instar larvae, showed better rhabdomere arrangement in Sp treated *park*^13^ flies as compared to untreated ones (Fig. 4a). Sp treatment resulted in presence of ~270 rhabdomeres close to mean wild type number of 290, an improvement over ~200 rhabdomeres observed in the mutants (Fig. 4b). The cytoprotective role of Sp was further evaluated through determination of mitochondrial function and amount of intracellular lipid peroxides quantified by measuring the levels of their decomposed carbonyl product malondialdehyde; the two components most vulnerable to oxidative damage due to increased mass of polyunsaturated fatty acids and respiratory activity in neurons. The *park*^13^ flies showed higher amounts of lipid peroxides which were considerably reduced upon Sp exposure (Fig. 4c). Similarly, Sp treatment resulted in maintained mitochondrial complex I activity which is found to be disrupted in PD patients and Parkinsonian models (Fig. 4d). As a result, the Sp-*park*^*13*^ flies presented healthier mitochondria sustaining higher membrane potential, as suggested by increased TMRE staining (Fig. 4e) and higher accumulation of mitochondrial mass, probably due to prevention of ROS-induced organellar damage (Fig. 4f). The Sp-flies, in-spite of possessing increased mitochondrial content, demonstrated reduced production of mitochondrial superoxides (Fig. 4g); thereby, promoting the observed neuronal recovery.

### Improved motor co-ordination in Scopoletin treated *park*^13^ flies

To test whether the improvement in neuronal abnormalities translated to rehabilitation of aberrant movement phenotypes including motion slowness, difficulties with gait and balance, we utilized the negative geotactic and phototactic behavior of *Drosophila* flies.

**Fig. 5:**
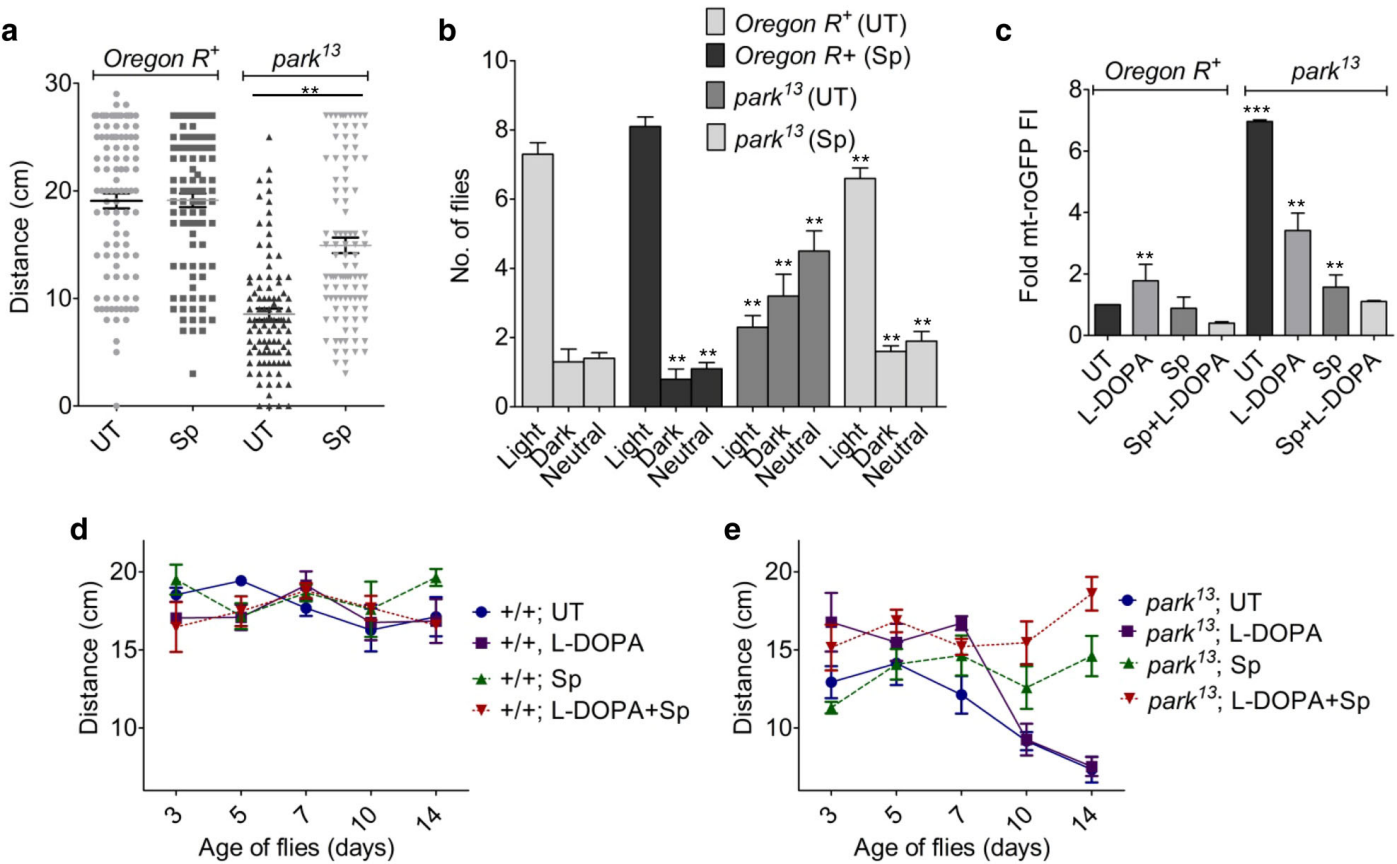
Sp results in recovery of motion and phototactic defects in Parkinsonian flies. **a** Fifteen days old *park*^13^ and wild type flies were subjected to negative geotaxis movement and the distance traveled through a vertical chamber was graphically represented as mean ± s.e.m.; *n* = 5, **P (*t*-test) <0.001. **b** The relative distribution or no net movement (Neutral) of wild type and mutant flies across the illuminated (Light) and darkened (Dark) arms of a Y-maze was observed and plotted as mean ± s.e.m.; *n* = 5, **P (*t*-test) < 0.001. **c** The fold change in mt-roGFP fluorescence intensities (FI) of the dopaminergic neurons in wild type and *park*^13^ flies exposed to 1 mM L-DOPA alone or in combination with 2.5 mM Sp, compared to untreated wild type control. Bars represents mean ± s.e.m.; *n* = 10, **P (*t*-test) < 0.001, ***P (*t*-test) <0.0001. **d**, **e** Temporal determination of negative geotactic movement of wild type (+/+) (**d**) or mutant (**e**) flies continuously fed with 1 mM L-DOPA, 2.5 mM Sp or combination. Each data point represent mean ± s.e.m.; *n* = 5, *P* < 0.001.

The wild type flies, irrespective of their treated conditions showed strong negative geotaxis through a vertical column. In contrast, the neuromotor dysfunction in *park*^13^ flies reflected locomotion defects where the mutant flies showed >50% compromised climbing activity compared to wild type. Sp treatment resulted in appreciable recuperation of the locomotor activity close to normal levels (Fig. 5a). Similarly, disoriented movement in *park*^13^ due to absence of photosensing capability was reasonably restored in Sp treated flies. The flies showed positive phototactic orientation quite similar to wild type pattern (Fig. 5b); hence, demonstrating efficaciousness of Sp in managing motor control abnormalities.

### Scopoletin alleviates toxicity associated with long term exposure to L-DOPA

L-DOPA has been the mainstay and most efficacious in management of PD phenotypes. However, its administration has been plagued by absence of long term benefits, oxidative toxicity being one of the major reasons thus, freezing the patients mid-sentence^39,40^. Although continuous treatment of *park*^13^ flies with L-DOPA reduced the inherent oxidative stress consistent with previous results, the flies still showed considerably higher levels of oxidative radicals (Fig. 5c). A similar observation was seen in L-DOPA treated wild type flies. The increased levels of L-DOPA dependent ROS was significantly reduced upon Sp exposure, both in wild type and *park*^13^ flies (Fig. 5c). The alleviation of L-DOPA toxicity by Sp was reflective of non-stasis in the recovery of motor abilities in *park*^13^ flies and the motility of the flies for extended period was comparable to the controls (Fig. 5e). L-DOPA alone contributed to strong recovery of motion phenotypes during initial phase, however, its effect lagged in aged flies which showed movement defects similar to untreated *park*^13^ mutants (Fig. 5e), probably due to dyskinesia usually associated with protracted L-DOPA exposure. The combination did not show any apparent deleterious effect in wild type controls (Fig. 5d), indicative of their potential usage in PD care.

## Discussion

The vulnerability of dopaminergic neurons to minor perturbations in redox equilibrium is enhanced by the oxidative by-products of dopamine metabolism, resulting in their selective loss in Parkinson’s disease. In the nigrostriatal tract system, dopamine undergoes autooxidation or oxidative deamination by monoamine oxidase-B, both of which aggravates with age, leading to increased production of peroxides and hydroxyl radicals^41^. In addition, age dependent accretion of dysfunctional mitochondria elevates stress level, which is exhibited by high levels oxidized products such as protein carbonyls and lipid peroxides^42^. The failure of the antioxidant system to negate the pathological rise in the oxidative species is exacerbated by reduction in levels of the major cellular nucleophile, GSH due to their covalent modification by dopamine quinone species^20,43–45^. Therefore, complementing the resistance of redox equilibrium to oxidative shift by supplementing the GSH levels has been considered to be one of the plausible approaches to contain the progressiveness of the disorder^46^.

A large number of antioxidant based strategies focusing on ascorbic acid, deferoxamine, cucurmin etc has been investigated for management of the PD symptoms but without any translatable benefits^47,48^; thus requiring elucidation of better alternatives with robust antioxidant capabilities to promote neuronal health in PD. Natural derivatives have been increasingly appreciated for their potency in prevention and management of neurodegenerative diseases^35,49,50^. *Convolvulus pluricaulis* extract is one of the most widely used neuropharmacological agent that significantly improved learning and memory in experimental models^34,51,52^. It has been employed in treatment of dementia, phobias and insomnia, and successfully tried in PD cases^53^. *C. pluricaulis* also has been found to ameliorate tau induced neurotoxicity in Alzheimer’s disease^54^. Our results suggest that *C. pluricaulis* majorly derives its neuroprotective properties through a robust antioxidant Sp.

Sp was able to restore cellular redox homeostasis under induced oxidative stress in mammalian cells and in vivo model, *Drosophila* where it ameliorated catalase and glutathione peroxidase deficiency. Further exposure of the Parkinsonian flies to Sp resulted in diseased flies to display more number of viable non-stressed dopaminergic neurons than the non-treated ones. Higher mitochondrial metabolism in the neurons result in generation of Complex I dependent hydrogen peroxide and neuronal membranes, rich in polyunsaturated fatty acids, are particularly vulnerable to oxidative stress due to their possession of unsaturated double bonds. Peroxidation of lipids disturbs membrane organization, causing functional loss of proteins and DNA, and are found elevated in PD patients where lipid peroxidation adducts contribute to early demise of the SNpc neurons. Mitochondria being the source and target of lipid peroxides accelerate the process^4,8,20^. Sp presented commendable recovery of the mutant flies from accumulated lipid peroxides and also induced parallel recovery of mitochondrial health; thus translating into better neuronal architecture observed in treated *park* flies. The potent neuro-protective ability of Sp was mainly derived from its ability to maintain glutathione levels in its oxidizable form, possibly by preventing its catabolism, though the exact chemical mechanism needs to be elucidated through further studies. The idea is supported by maintenance of functional GSH/GSSG levels by Sp in BSO and neurotoxin treated cells as compared to untreated ones which showed a significant decline in the antioxidant ratio probably, due to enhanced formation of glutathione–quinones^43^; hence, allowing Sp to effectively circumvent the ROS induced toxicity associated with the prevailing therapies.

The considerable recovery of the dopaminergic neuronal clusters in the *park* flies ensued better negative geotactic behavior in the flies and prolonged their life span, suggesting improvement in motor coordination dysfunction associated with PD. The flies solely administered with L-DOPA performed better during initial exposure after which their progress declined, consistent with the reported pattern of “wearing-off” in L-DOPA administered PD patients^39,55^. However, Sp-L-DOPA combination showed consistent negative geotaxis even during the “wearing-off” phase, representing substantial alleviation of L-DOPA toxicity and promotion of enhanced mitochondrial and cellular health in dopaminergic neurons.

Notwithstanding the strides achieved in genetic manipulation and regenerative technologies, L-DOPA or compounds related to dopamine metabolism such as MAO-B inhibitors remain as the major modes of palliative care for PD patients^39,56^. However, these regimens have been plagued with long term drug toxicity leading to retarded neutrite growth, mitochondrial dysfunction, and oxidative stress^18^. Enhancement of the intracellular reducing equivalents such as glutathione, to increase the cellular competence to electrophiles holds promise to impede the progression of the disorder and had been clinically effective in PD patients. Standalone or combinatorial regimens designed around potent bio-absorptive reductants, elevating the efficiency of palliative treatment by decreasing or delaying disease progression, will be a welcome step for further trials and those affected by the disease.

## Material and methods

### Cell culture

HeLa (ATCC Number: CCL-2) and SHSY-5Y (ATCC Number: CRL-2266) cells, STR profiled and free from any contamination, were sourced from National Center for Cell Science repository. The cells were cultured in Eagle’s minimum essential medium (ThermoFisher Scientific) or DMEM F-12 (ThermoFisher Scientific) respectively, supplemented with 10% Fetal Bovine Serum (Gibco) and 1% penicillin–streptomycin (ThermoFisher Scientific), in a humidified incubator maintaining 5% CO_2_ at 37 °C. All assays were performed in cells maintained at ~60% confluency.

### Fly strains

UAS-GFP (Bloomington Stock #6874), TH-Gal4 (Bloomington Stock #8848), CatalaseRNAi (Bloomington Stock #34020) and PHGPx (Bloomington Stock #33939), were obtained from Bloomington stock center, Indiana, along with *park*^13^ (Bloomington Stock #79210) as the Parkinsonian disease model. *Oregon R*^*+*^ was used as the wild type control line for all experiments. The stocks were maintained in 24 °C incubator. The genetic combinations used in the assays were generated by standard genetic crosses.

### ROS measurement

Oxidative stress was induced by exposing 1.5 × 10^5^ HeLa cells seeded overnight, to 10 µM 3-AT for 15 min at 37 °C; followed by treatment with 250 µM of the Sp for 15 min. Cellular glutathione levels were depleted by co-exposing the cells to 50 µM BSO for 15 min. 1.2 × 10^5^ SHSY-5Y cells were subjected to similar treatment as above except 1 mM MPP^+^ (for 24 h) was used to induce ROS. Post-treatment, the cells were washed with 1X phosphate-buffered saline and stained with 15 μM DCFDA-H2 or 5 μM MitoSOX for 15 min at 37 °C. The estimation of total cellular GSH and GSSG levels was performed through GSH/GSSG-Glo™ Assay Kit (Promega) as per manufacturer’s instructions. GGT and GCL activity was determined through GGT-Activity Colorimetric Assay Kit (Merck Sigma-Aldrich) and GCL Assay Kit (Real-Gene Labs) respectively, following the protocol detailed by the manufacturer. The observed activities were normalized over the total protein content estimated through Quick Start™ Bradford reagent (Bio-Rad). Fluorescence/luminescence/absorbance intensities at appropriate wavelengths were quantified in Synergy H1 microplate reader (version 2.09.1). In case of *Drosophila*, neuronal redox balance was altered by driving CatalaseRNAi or PHGpxRNAi, along with roGFP by elav-Gal4 to deplete the respective antioxidant enzymes and express the redox biosensor. The first instar larvae taken 24 h AEL (after egg laying), was orally administered with 2.5 mM Sp and fluorescence measurement in brains of eclosed adults were performed as mentioned above. To test the effect of Sp in paraquat-induced oxidative stress model, seven days old wild type flies, constitutively expressing mitochondrial (mt)-roGFP, were exposed to 10 mM paraquat for 24 h and then co-treated with 2.5 mM Sp for 24 h, prior to roGFP fluorescence estimation. In case of *park*^13^, mt-roGFP was expressed in dopaminergic neurons by TH-Gal4 for estimation of redox state in twenty days old adult flies either individually treated with 2.5 mM Sp or co-treated with 1 mM L-DOPA from 10 days AEL. The concentrations of Sp and L-DOPA were kept similar for all experiments unless otherwise mentioned. Each replicate constituted of twenty flies. Equivalent numbers of age-matched unsegregated flies were used for all experiments except survival assay.

### Cellular viability

Optimization of Sp concentration and its cytotoxicity parameters were established by treating of 1.5 × 10^5^ HeLa or 1.2 × 10^5^ SHSY-5Y cells with serial concentrations of the Sp (100 µM–1 mM) for 15 min or conducting a time-course (5 min–48 h) using 250 µM Sp, followed by MTT assay as per standard protocol (Thermo Fisher Scientific). The absorbance was taken at 540 nm in Synergy H1 microplate reader (version 2.09.1). The rescue of cell viability by Sp was measured by following a similar treatment regimen as described above for ROS profiling of the cells. Recovery from ROS-induced toxicity was tested through MTT assay, in HeLa and SHSY-5Y cells pre-exposed to 10 µM 3-AT for 15 min or 1 mM MPP^+^ for 24 h respectively, followed by 250 µM 15 min Sp treatment.

### Confocal imaging

Tyrosine hydroxylase (TH)-Gal4 driven UAS-GFP lines, labeling the neurons with active dopamine synthesis pathway, were crossed with *park*^*13*^ flies and progenies were exposed to Sp from 10 days AEL. Twenty days old adult brains from the resultant F1 progeny carrying the genotype *TH* > *Gal4; park*^13^, were imaged under Sp treated and untreated conditions to assess the integrity of dopaminergic neurons. Rhabdomere arrangement was visualized in third instar (120 h AEL, Sp fed for 48 h) larval eye imaginal disc by labeling with anti-elav antibody (9F8A9, DSHB). Image analysis was performed through Zeiss 510 META confocal microscope and processed with LSM image browser. Rhabdomere quantification was done through ImageJ. Images were minimally processed for brightness-contrast adjustments using Adobe Photoshop 7.0.

### Assessment of mitochondrial function

Ten numbers of anesthetized twenty day old adult flies were dissected in cold *Drosophila* Schneider’s Medium (DSM). The dissected brains were incubated in 100 nM TMRE solution (Thermo Fisher Scientific) in DSM for 10 min and relative fluorescence intensity was quantified by top excite-read method through Synergy H1 reader. For estimation of mitochondrial mass, the fly brains were stained with 20 µM N-Nonyl Acridine Orange for 15 min and fluorescence intensity was determined as above. Quantification of mitochondrial superoxide was similarly executed except that the samples were stained with 5 µM MitoSox^TM^ Red (Thermo Fisher Scientific). Activity of complex I was quantified based on the protocol described by Spinazzi et al.^57^ Mitochondria, isolated using mitochondria isolation kit (Sigma), were incubated in phosphate buffer containing 1% DOC and 2 mM sodium azide. After 15 min, mitochondria were incubated with 1 mM NADH. The reaction was initiated with the addition of 60 μM ubiquinone and the decrease in absorbance was recorded at 340 nm for 2 min.

### Lipid peroxidation

The brains of 20 days old wild type and mutant flies, Sp treated or kept untreated as mentioned above, were dissected and homogenized in DSM. Then, 1% thiobarbituric acid was added to the clarified homogenate and incubated at 90 °C for 1 h, followed by addition of 250 μL of n-butanol. The solution was centrifuged at 16,000 × *g* for 2 min and the supernatant was used to measure absorbance at 530 nm.

### Behavioral assays

Phototactic Assay was conducted to evaluate optic nerve degeneration, employing Y-shaped glass maze with lightened, dark and light neutral ends. Ten flies per replicate were introduced in the neutral end and their migration into each branch was observed. Assessment of neuromuscular coordination was performed by evaluating the ability of the flies to ascend a vertical graduated plastic cylinder. Each set consisted of five flies kept at the bottom of the tube. The tube was gently shaken and the final distance traveled within 10 s was recorded. 20 days old flies, Sp and/or L-DOPA fed from 10 days AEL, was used in all the experiments.

### Survival assay

Measurement of life span in wild type and *park*^*13*^ flies was essentially performed as per established protocols^58^. Briefly, 100 age-matched male and female flies were segregated in separate vials and transferred to fresh vials during the experimental period every five days. Dead flies were recorded at the time of each transfer process. Flies exiting the experiment were censored and not recorded as dead. Data was subjected to Kaplan Meier analysis in Graphpad Prism.

### Statistical analysis

The sample size possessing sufficient statistical power for each experiment is provided in the figures or mentioned under respective sub-headings in the methods section. Data are representative of minimum three independent experiments and the number of biological replicates is quoted in the figure legends. Mean and standard error was calculated for all quantitative experiments. Statistical significance was determined using two-tailed Student’s *t* test through GraphPad Prism5 software unless otherwise mentioned. The *P* values have been represented by comparing the data pairwise with wild type untreated controls and are mentioned in figure legends. Statistical significance was specified as *P < 0.01, **P < 0.001, or ***P < 0.0001.

## Supplementary information

Supplementary Figure S1

Supplementary Figure Legends
