# Supplementary figures and images for: Enhanced accumulation of reduced glutathione by Scopoletin improves survivability of dopaminergic neurons in Parkinson’s model
Source: Cell Death Dis. 2020 Sep 10;11(9):739. doi: 10.1038/s41419-020-02942-8 (PMC7484898; doi:10.1038/s41419-020-02942-8)

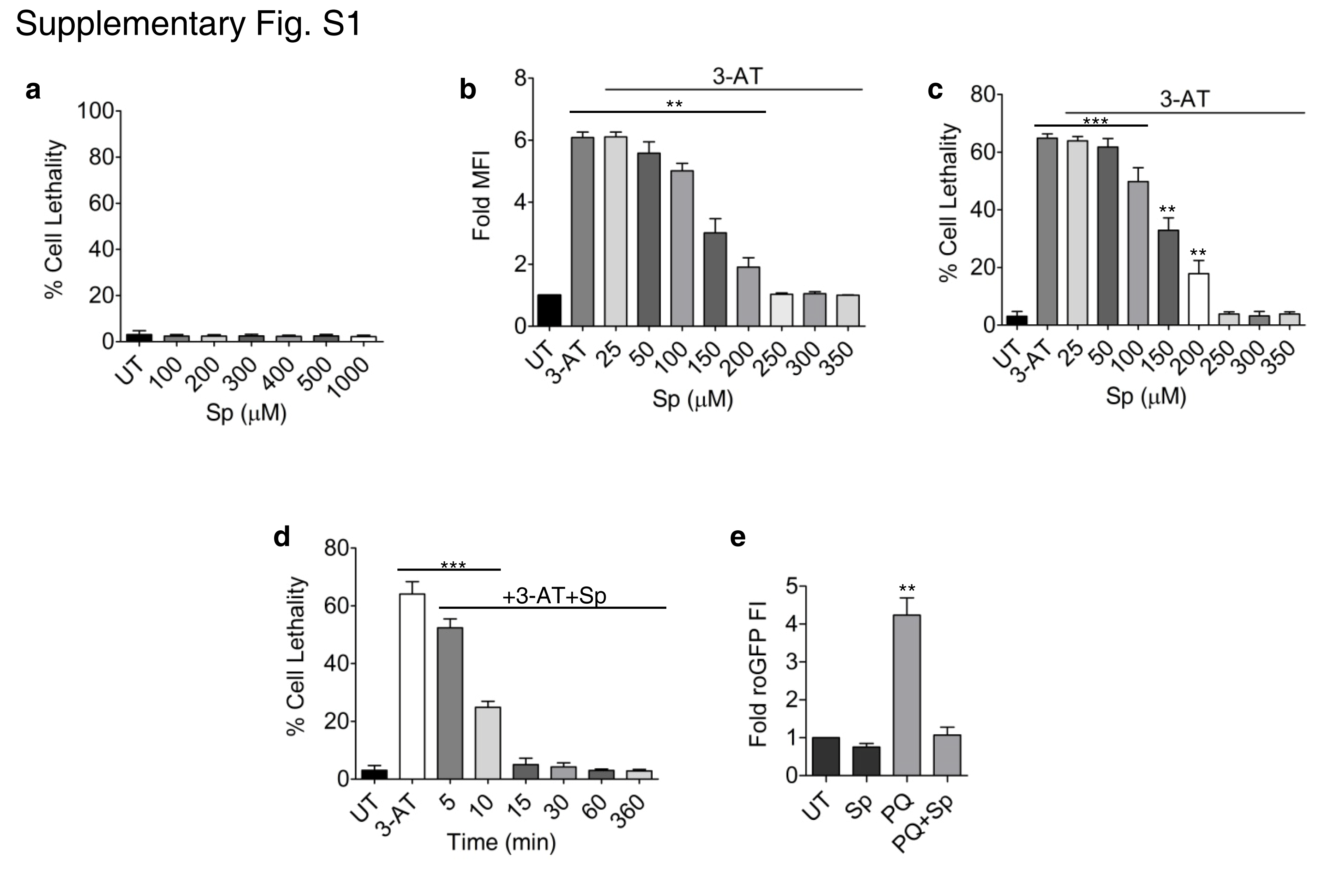

Supplement: Supplementary file 1 — Supplementary Figure S1 [file 41419_2020_2942_MOESM1_ESM.tif]
