# Supplementary material for: Enhanced accumulation of reduced glutathione by Scopoletin improves survivability of dopaminergic neurons in Parkinson’s model
Source: Cell Death Dis. 2020 Sep 10;11(9):739. doi: 10.1038/s41419-020-02942-8 (PMC7484898; doi:10.1038/s41419-020-02942-8)
Supplement: Supplementary file 2 — Supplementary Figure Legends [file 41419_2020_2942_MOESM2_ESM.docx]

**Supplementary Figure Legends:**

**Fig. S1:**

**(a)** 1.5 x 10^5^ HeLa cells treated with increasing concentrations of Sp for 15 min, were subjected to MTT assay. Bars depict mean ± s.e.m.; n = 8. **(b)** HeLa cells pre-treated with 3-AT, were exposed to increasing concentrations of Sp to determine the optimal Sp concentration where ROS scavenging activity is observed. The cells were stained with DCFDA and mean fluorescence intensity (MFI) was measured and represented as fold change over untreated samples. Bars depict mean ± s.e.m.; n = 3, ***P*(*t*-test) <0.001. **(c)** HeLa cells subjected to above treatment were assessed for viability through MTT assay and plotted as mean ± s.e.m.; n = 8, ***P*(*t*-test)<0.001, ****P*(*t*-test)<0.0001. **(d)** HeLa cells were subjected to time course experiment by pre-exposing them to 10 µM 3-AT for 15 min, followed by 250 µM Sp co-treatment for indicated time periods. The survivability of cells were estimated by MTT assay and data was plotted as mean ± s.e.m.; n = 8, ****P*(*t*-test)<0.0001. **(e)** Wild type *Drosophila* flies continuously exposed to Sp for 10 days from 120 h AEL, were treated with 10 mM paraquat (PQ) for 24 h, followed by quantification of oxidative stress through roGFP flourescence intensity (FI). Data is represented as mean ± s.e.m.; n = 6, ***P*(*t*-test)<0.001.
